# Oropharyngeal and otorhinological changes in end stage renal patients undergoing hemodialysis

**DOI:** 10.4317/jced.58292

**Published:** 2021-07-01

**Authors:** Ola M. Ezzatt, Mohamed G. Hamed, Yasmine Gamil

**Affiliations:** 1 Associate professor in department of Oral Medicine, Periodontology, Oral Diagnosis and Radiology, Faculty of Dentistry, Ain Shams University, Cairo, Egypt; 2 Assistant professor in department of Otorhinolaryngology, Faculty of Medicine, Helwan University and ENT consultant in Arayah Hospital in Maadi, Cairo, Egypt; 3 Lecturer in department of Oral Medicine, Periodontology and Oral Diagnosis. Faculty of Dentistry, Modern University of Information and Technology (MTI). Cairo, Egypt

## Abstract

**Background:**

The study aimed to assess oropharyngeal and otorhinolaryngological changes in end stage renal disease (ESRD) patients undergoing hemodialysis and correlate the findings to renal functions.

**Material and Methods:**

This case-control study compared oral and otorhinolaryngological findings in 85 patients with (ESRD) on maintenance hemodialysis to age and sex matched 85 healthy controls. Frequencies of findings were calculated and compared and correlation between biochemical and the oral health parameters in case group was determined using T-test, chi-square and Pearson correlation test (significance were set at *P*<0.05).

**Results:**

The frequency of oral signs and mucosal symptoms were significantly higher among ESRD compared to healthy controls. Dry mouth (34.12%), bad odour (32.94%), increased tongue coating (50.59%) and pale mucosa (45.88%) were the most commonly reported. Otorhinolaryngological findings was higher in cases than in controls, with otomycosis (10.59%) and allergic rhinitis (5.88%) being the most frequent findings. Serum creatinine and blood urea mean levels were higher in ESRD patients with oral and otorhinolaryngological findings compared to those without findings.

**Conclusions:**

Oral and nasal manifestations in patients with ESRD on maintenance hemodialysis were significantly higher in comparison to healthy individuals and were related to their serum creatinine and blood urea mean levels.

** Key words:**Chronic kidney disease, renal dialysis, Oral manifestation, nasal, case control, Egypt.

## Introduction

The sequalae of primary renal disease or systemic diseases with renal involvement with loss of kidney function is a final syndrome called End Stage Renal Disease (ESRD), causing manifestations involving virtually every system and characterized by a profound alteration of water, electrolyte, and acid-base homeostasis, as well as retention of uremic toxins, especially protein catabolism nitrogen waste products (1).

Various signs and symptoms are presented in patients with ESRD, reflecting the improper kidney’s functions (2), or adverse drug reactions in every organ system (3).

A wide variety of oropharyngeal manifestations and symptoms have been documented in these patients by several authors including halitosis, xerostomia, periodontitis, dysgeusia, candidiasis, parotitis, abnormal lip pigmentation, burning mouth sensation and ulcerations (4-6). Determination and identification of these changes in the oral cavity will improve the quality of life in these patients. Furthermore, it is important to consider patients with ESRD on maintenance hemodialysis (HD) as a candidate of renal transplant in the future. Since the availability of the transplant is unpredicTable, and when it occurs the time interval of pretransplant preparation is too short to manage all the existing oral infections in HD patients, therefore, it is worth to evaluate and maintain an acceptable oral health status of these dialysis patients at least until successful transplantation (7).

The most commonly analyzed abnormalities in head and neck area in patients with chronic kidney diseases (CKD), were sensorineural hearing loss, epistaxis, candidiasis, halitosis, xerostomia, dysgeusia, lip and thyroid cancers (8).

The study aimed to investigate the prevalence of oropharyngeal changes and otorhinolaryngological changes in Egyptian hemodialysis patients compared to healthy individuals; and to assess possible association between subjective symptoms and objective clinical findings and serum urea and creatine levels.

## Material and Methods

-Study design: It is a case-control observational single center study.

Sample size estimation: It was anticipated that the prevalence of oral lesions cases among chronic kidney diseases case group was 96.7% and that the prevalence in the healthy control group was 16.7% as reported by Oyetola *et al*. (9) The minimum required sample size per group was calculated to be 71 participants using Epitools web-based application at (https://epitools.ausvet.com.au/casecontrolss?page=case-controlSS) assuming relative risk of (5.8) with the standard normal values set at 0.05, a power of 90 % and a case–control ratio of 1:1. Considering about 20 % sample attrition; we included 170 participants (85 patients with chronic kidney diseases undergoing hemodialysis and 85 matched healthy controls).

-Subjects: A total of 170 adult participants of both genders above 18 years were included in the study. 85 patients were recruited for cases group from consecutive patients diagnosed by end-stage renal failure regardless of the etiology of the condition and regularly attending the nephrology hemodialysis department for more than three months at (Arayah Hospital in Maadi, Cairo, Egypt. Participants with history of smoking (cigarette, pipe or water pipe) or alcoholic patients in the last 10 years were excluded.

The control group populations were randomly recruited from (Out-patient dental clinic and ENT clinic at (Arayah Hospital in Maadi, Cairo, Egypt) to include total 85 clinically healthy participants, with no history of kidney disease, or any other chronic debilitating illness, or habit of smoking or drinking, and/or not receiving any medication that could affect oral health. Participants with history of excessive or chronic noise exposure, congenital ear deformities, otological trauma or surgery have been excluded from both groups.

-Ethics approval and consent to participate: This study was conducted in full accordance with the World Medical Association Declaration of Helsinki. The brief explanation of the purpose of the study, the optional contribution with confidentiality and sole use of the information for the mentioned purpose was ensured. All subjects were informed about the procedure and their agreements were taken by signing the informed consent.

-Data collection: For eligible participants full medical records were reviewed including last measured serum creatinine and urea levels in cases group. Data recording and examination were performed at the bed side during hemodialysis session for patients’ convenience.

Detailed intraoral examination for the entire oral and pharyngeal mucosal surfaces. Signs and symptoms identification were objectively searched for, and /or reported by the patients for any mucosal changes using simple disposable dental examination instruments. Oropharyngeal lesions were diagnosed and documented according to the history and clinically accepted criteria based on the WHO Guide to Epidemiology and Diagnosis of Oral Mucosal Diseases. Otorhinolaryngological dysfunctions were assessed and recorded by the ENT specialist (ear examination by inspection, palpation and otoscopic examination. Hearing assessment by tuning fork test and suspected hearing loss patients were subjected to pure tone audiometry and tympanometry. Nasal, pharyngeal and laryngeal examination was performed using simple outpatient clinic tools.

-Statistical analysis: Data was collected, tabulated and then subjected to the statistical analysis using the Statistical Package for Social Sciences (SPSS) (version 13.0 for windows, SPSS Inc., Chicago, IL, USA). The qualitative presented as numbers and percentages. The oral and otorhinolaryngological health data from the dialysis patients was compared to those from matched control group using independent Student t-tests, chi-square tests or Fisher’s exact tests when appropriate. The Pearson correlation was determined between biochemical and the oral health parameters in case group. All levels of significance were set at *P*<0.05.

## Results

One hundred and seventy subjects participated in the study, 86 (50.59%) males and 84 (49.41%) females with age ranged from 18-78 years. The participants in cases and control group were matched for age and sex distribution with no statistically significant differences (*p*=0.53). The duration of the end stage renal diseases in subject undergoing hemodialysis ranged from 2 to 120 months and it was significantly correlated to patient age (*P*-value=0.026).

The most common cause of (ESRD) among patients was chronic glomerulonephritis (54.12%), followed by diabetic nephropathy (16.47%), while the remaining subjected reported other causes like hypertensive nephropathy or the cause was unknown. Combined anemia, diabetes and hypertension was reported in 31.76% of cases and 51.76% of patients were under antihypertensive medications while 43.53% were on calcium-carbonate supplementation. Considerable percentages of patients were taking antiplatelet, antidiabetic, multivitamins or diuretics (Table 1).

**Table 1:**
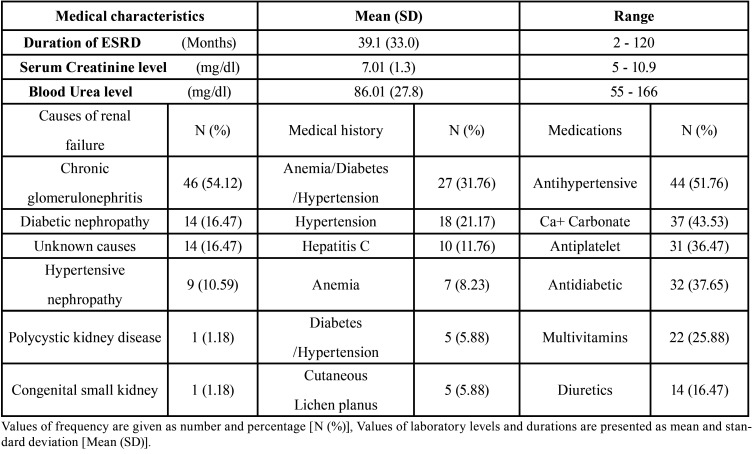
Medical characteristics and history of patients with end stage renal diseases undergoing renal dialysis patients (ESRD).

**Figure 1:**
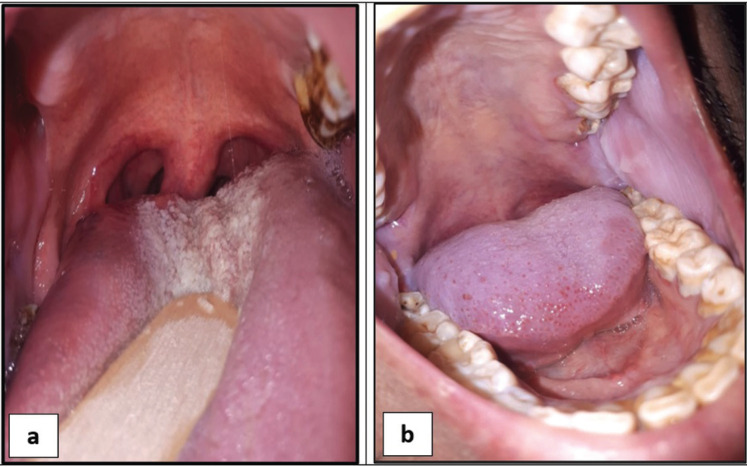
Photographs of oral findings in hemodialysis group of patients including increased tongue coating (a) and pale mucosa (b).

The frequency of oral signs and mucosal symptoms were higher among ESRD compared to healthy control subjects and the differences were statistically significant (Table 2). Dry mouth (34.12%) and bad odour (32.94%) were the most commonly reported symptoms followed by unpleasant taste and burning sensation, while increased tongue coating (50.59%) and pale mucosa (45.88%) followed by petechiae/ecchymosis, uremic stomatitis, dry fissured tongue and mucosal hyperpigmentation were the most frequent oral findings among ESRD (Fig. 1).

**Table 2:**
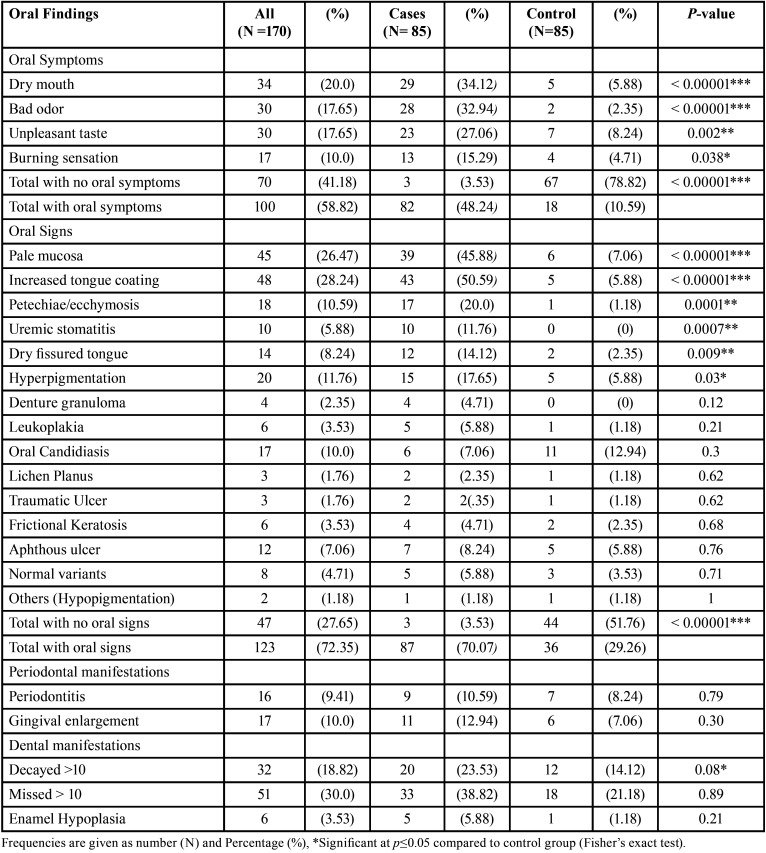
Comparison of frequency of oral signs, mucosal symptoms, periodontal and dental manifestations among end stage renal diseases patients undergoing hemodialysis (ESRD) and healthy control subjects.

Prevalence of otorhinolaryngological and pharyngeal signs and dysfunctions were higher in cases than in control group however the differences were not statistically significant except with nasal findings (P-value=0.025). (Table 3). Bilateral otomycosis (10.59%) and allergic rhinitis (5.88%) were the most commonly reported ear and nasal findings respectively while 28.24% of subjects were difficult to be examined for laryngeal signs.

**Table 3:**
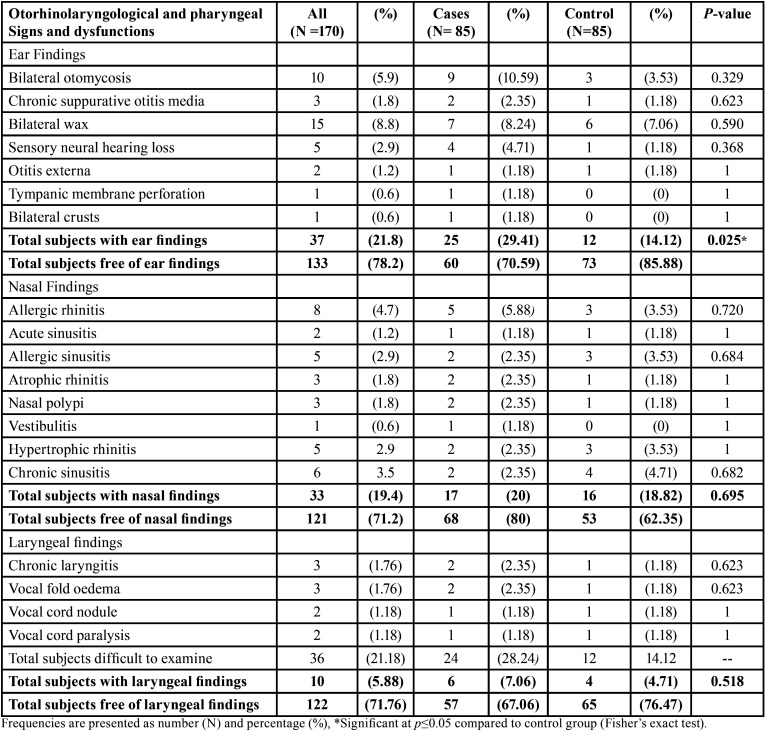
Comparison of frequency of pharyngeal and otorhinolaryngological signs and dysfunctions among end stage renal disease patients undergoing hemodialysis (ESRD) and healthy control subjects.

**Figure 2:**
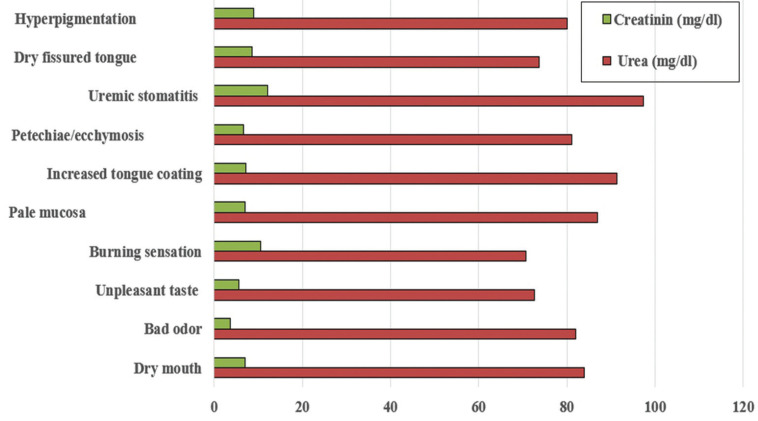
A bar graph showing comparison between serum and creatinine levels among ESRD patients manifested with oral signs and symptoms.

Uremic stomatitis was associated with highest urea and creatinine level, while bad odour was frequent among those with low serum creatinine levels and burning sensation among those with lowest blood urea levels (Fig. 2).

Serum creatinine and blood urea mean levels were higher in ESRD patients with oral and otorhinolaryngological findings compared to those without findings however the difference was significant only regarding creatinine level (Table 4).

**Table 4:**
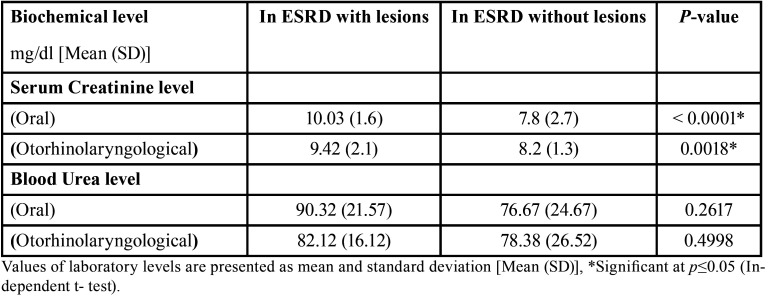
Serum creatinine and blood urea level relation to presence of oral or otorhinolaryngological findings.

## Discussion

The present study revealed significantly higher prevalence of oral symptoms (48.24%) and lesions (70.07%) as well as otorhinolaryngological findings (29.41%) in ESRD patients undergoing hemodialysis in comparison to healthy individuals. These findings were generally reported in other studies (8-10) and among other populations (11), which could be related to the medical, psychological, or socioeconomic characteristics of these patients (12).

Our data revealed highest prevalence of dry mouth (34.12%) among the reported oral symptoms which was in agreement with the 32.9% to 68% Figures reported in other stu¬dies of dialysis patients (13-15). Dry mouth in ESRD patients may be a multifactorial phenomenon; mouth breathing and medication use (13), water restriction, low saliva flow, minor salivary glands parenchymal fibrosis and atrophy (15), being identified factors (14).

Bad odor and unpleasant taste as frequently reported symptoms may be attributed to increased levels of urea in the blood and also in the saliva, which turn into ammonia by the action of urase-splitting oral organism and exaggerated by xerostomia (16). Contrary to previous researches that showed removal of urea and other toxin via dialysis results in improved taste function (17,18). This, could be related to low animal proteins intake, low zinc levels, increased phosphate concentrations or changes in saliva pH, which led to unpleasant or metallic taste. The occurrence of burning sensation reached 15.29% of all patients. This result was fairly similar to that reported by Nandan *et al*. (13).

Our results revealed that the most frequent oral lesions among ESRD patients were pale oral mucosa (45.88%) and increased tongue coating (about 50.59%), gingival enlargement and uremic stomatitis. These results were higher than that reported by de la Rosa-Garca (19), and Chuang *et al*. (20). and lower than that reported by Anuradha *et al*. (21).

Increased tongue coatings probably caused by retention of desquamated epithelial cells and by volatile sulfurous compounds produced by anaerobic bacteria on the tongue surfaces. Poor oral hygiene, low saliva flow and even emotional condition of the dialysis patients have an impact on its occurrence.

The appearance of pale oral mucosa in ESRD patients explained mainly by anemia caused by erythropoietin and folic acid deficiencies (22). Pale mucosa has also been associated to malnutrition (23). Moreover, the yellowish mucosa discoloration may be caused by urocromo pigments (22).

Uremic stomatitis was found on 11% of ESRD patients. The lesion has been described as localized or generalized burning oral mucosa with erythematous areas covered by a grayish pseudomembranous exudate leaving, on removal, an intact (type I) or ulcerated (type II), often coexisting with candidiasis (24). Its etiology is unknown. It has been considered the reaction to an irritant; possibly ammonia compounds derived from urea hydrolysis by salivary urease, whenever saliva urea concentration exceeds 180 mg/dl (24). Uremic stomatitis was associated with highest serum urea and creatinine level in our study probably as a result of a high urea concentration in saliva, and its conversion to ammonia However, the lesion considered uncommon because of usually earlier dialysis therapy and that is why we found only few cases, just starting dialysis therapy, with oral lesion matching uremic stomatitis description.

Salivary and serum creatinine and urea levels in patients with chronic kidney disease are positively correlated which could be responsible for the complaints of dry mouth (2), mouth odour or uremic breath (20), as well as tongue coating and other oral complications in these patients (19). However, in the present study bad odour was most frequent among those with low serum creatinine levels and burning sensation among those with lowest blood urea levels. additional possible causes are changes in saliva pH, which might explain a metallic or unpleasant taste (25).

Otolaryngologic clinical manifestations, in comparison to the control group were non-significant except for the nasal findings which was more in the test group. Krajeweska *et al*. (2020) (8) and Peyvandi (2011) (26)also found that patients with CKD are prone to develop oropharyngeal candidiasis or rhino-cerebral mucomycosis, taste and smell changes, phonatory and vestibular dysfunctions and had higher prevalence of sensory neural hearing loss.

The study was limited by single center sampling and lack of salivary biochemical analysis, however frequent oropharyngeal changes and otorhinolaryngological dysfunctions was identified among this cohort of end stage renal disease patients undergoing with controlled socioeconomic confounding factors. Implementing therapeutic and preventive measures for those patients regarding these findings would be clinically relevant.
